# Exposure to Ozone Modulates Human Airway Protease/Antiprotease Balance Contributing to Increased Influenza A Infection

**DOI:** 10.1371/journal.pone.0035108

**Published:** 2012-04-09

**Authors:** Matthew J. Kesic, Megan Meyer, Rebecca Bauer, Ilona Jaspers

**Affiliations:** 1 Center for Environmental Medicine, Asthma, and Lung Biology, University of North Carolina Chapel Hill, North Carolina, United States of America; 2 Curriculum in Toxicology, University of North Carolina Chapel Hill, North Carolina, United States of America; 3 Department of Pediatrics University of North Carolina Chapel Hill, North Carolina, United States of America; 4 Department of Microbiology and Immunology University of North Carolina Chapel Hill, North Carolina, United States of America; Johns Hopkins University - Bloomberg School of Public Health, United States of America

## Abstract

Exposure to oxidant air pollution is associated with increased respiratory morbidities and susceptibility to infections. Ozone is a commonly encountered oxidant air pollutant, yet its effects on influenza infections in humans are not known. The greater Mexico City area was the primary site for the spring 2009 influenza A H1N1 pandemic, which also coincided with high levels of environmental ozone. Proteolytic cleavage of the viral membrane protein hemagglutinin (HA) is essential for influenza virus infectivity. Recent studies suggest that HA cleavage might be cell-associated and facilitated by the type II transmembrane serine proteases (TTSPs) human airway trypsin-like protease (HAT) and transmembrane protease, serine 2 (TMPRSS2), whose activities are regulated by antiproteases, such as secretory leukocyte protease inhibitor (SLPI). Based on these observations, we sought to determine how acute exposure to ozone may modulate cellular protease/antiprotease expression and function, and to define their roles in a viral infection. We utilized our *in vitro* model of differentiated human nasal epithelial cells (NECs) to determine the effects of ozone on influenza cleavage, entry, and replication. We show that ozone exposure disrupts the protease/antiprotease balance within the airway liquid. We also determined that functional forms of HAT, TMPRSS2, and SLPI are secreted from human airway epithelium, and acute exposure to ozone inversely alters their expression levels. We also show that addition of antioxidants significantly reduces virus replication through the induction of SLPI. In addition, we determined that ozone-induced cleavage of the viral HA protein is not cell-associated and that secreted endogenous proteases are sufficient to activate HA leading to a significant increase in viral replication. Our data indicate that pre-exposure to ozone disrupts the protease/antiprotease balance found in the human airway, leading to increased influenza susceptibility.

## Introduction

Influenza A virus is responsible for the seasonal epidemics and reoccurring pandemics, which represents a worldwide threat to global public health [1]. It is a major cause of morbidity and mortality worldwide, as during the recent H1N1 pandemic. In the U.S. alone, over 100,000 individuals are hospitalized and over 20,000 people die every year due to influenza virus infection and related diseases [2], [3]. Despite large-scale efforts in vaccination and antiviral therapies, the morbidity and mortality rates associated with influenza infections have not significantly changed in recent years [4], [5]. In the context of potentially pandemic respiratory viral infections, it is important to identify molecular targets/pathways for therapeutic intervention to protect susceptible sub-populations. Epidemiologic studies show that exposure to inhaled oxidants such as cigarette smoke, diesel exhaust, and ozone can modulate immune function and increase susceptibility to respiratory viral infections [6], [7], [8], [9], [10], [11]. Despite the extensive study into different factors influencing susceptibility to infection, the mechanism(s) by which inhaled oxidants modify viral pathogenesis are very complex and have yet to be fully elucidated.

The ability of oxidants to cause lung injury/dysfunction is dependent, in part, on the delicate equilibrium that exists between oxidants and antioxidants. Oxidative stress is caused by an imbalance between the production of reactive oxygen species (ROS) and the body's ability to readily detoxify reactive intermediates. Ozone is one of the most abundant components of air pollution in urban areas, and has been shown to be a potent inducer of oxidative stress causing airway inflammation and increased respiratory morbidities [8], [9], [12]. The Mexico City Metropolitan Area (MCMA), one of the most densely populated areas in the world, experiences high levels of air pollutants such as environmental ozone and particulate matter (PM) [13], [14], [15]. The MCMA is located 2240 m above sea level and is surrounded by mountains. Due to this geographical location, there is less available oxygen, making combustion less efficient, which produces more polycyclic aromatic hydrocarbon (PAH) pollutants. For these reasons, Mexico City receives higher levels of environmental ozone and various other types of photochemical smog [13]. It was here that the first influenza pandemic of the 21^st^ century emerged in March 2009. It was responsible for an estimated ∼258,698 laboratory confirmed cases and roughly ∼1,370 deaths by December 2009 [1], [16], [17]. The normal “Flu Season” occurs during the colder half of the year in each hemisphere [18]. Interestingly, this outbreak emerged during the dry and warmer months when ozone levels were significantly higher [19], [20].

Possible mechanisms by which oxidative stress alters the airway environment leading to broadened cellular tropism and/or susceptibility to viral infections have been proposed. [21], [22], [23] The relationship between oxidative stress and the cellular protease/antiprotease balance has been considered to be a major contributor in the development of numerous airway pathologies [24]. Early studies demonstrated that the cleavage of influenza HA is essential for viral penetration into host cells and that this mechanism was mediated by cellular trypsin-like serine proteases. These proteases, in turn, are regulated by mucus antiproteases, such as SLPI and α_1_-antitrypsin (A1AT) [25], [26], [27], [28], [29]. Recent studies have identified specific cellular proteases and antiproteases that may be involved in influenza infection, which include transmembrane protease serine 2 (TMPRSS2), human airway trypsin-like protease (HAT), and secretory leukocyte proteinase inhibitor (SLPI) [30], [31], [32], [33], [34]. The expression of these proteases in the lung are necessary for cleavage of the viral HA surface protein, thus allowing viral fusion and entry into the host cell. Studies have shown a correlation between inflammation and oxidative stress which alters expression of these proteases and antiproteases [29], [35]. Specifically, HAT has been shown to be released into the airway fluids under inflammatory conditions, particularly in asthmatics [31], [36]. In addition, a murine study showed SLPI expression was suppressed and protease expression was increased in Nrf2-deficient mice which led to increased inflammation, further demonstrating a relationship between oxidative stress and protease expression [37]. Oxidative stress derived from cigarette smoke or addition of reactive oxygen intermediates has been shown to decrease antiprotease activity [35], [38] and a murine study demonstrated that decreased antiprotease expression increased influenza infectivity [39]. The imbalance of protease/antiprotease expression caused by oxidative stress has been attributed to both an increase in inflammatory cells, which release proteolytic enzymes capable of destroying lung tissue, and a functional deficit of antiproteases due to oxidation of their active site [35], [40]. In addition to influenza, studies have shown that regulated proteolysis is required for the spread/propagation of many human viruses, including Human immunodeficiency virus (HIV), Nipah, Ebola, severe acute respiratory syndrome coronavirus (SARS-CoV), and metapneumoviruses [41], [42], [43], [44], [45].

Although it has been shown that oxidative stress increases severity of viral infections, the exact mechanism as to how and why this happens and the role of ozone exposure in these responses are not fully understood. While the deleterious health effects of both ozone and influenza have been well documented, few studies have looked at the effects of ozone in the context of an influenza infection. Herein, we used our established cell culture model of differentiated human nasal epithelial cells (NECs) [46], exposed them to 0.4ppm of ozone, and determined the effects on susceptibility to an influenza A infection. We show that there is a delicate balance of proteases and antiproteases present in the human airway surface liquid. Additionally, we found exposure to ozone disrupts this equilibrium by enhancing protease secretion while inhibiting antiprotease expression leading to increased susceptibility to viral infection. We directly show that ozone increases soluble protease expression and that they are functional for cleaving the viral HA protein and activation of the intact influenza virion.

## Materials and Methods

### Ethics Statement

Primary human nasal epithelial cells (NECs) were obtained from healthy adult volunteers. This protocol was approved by the University of North Carolina School of Medicine Institutional Review Board for Biomedical Research. In addition, written informed consent was provided by each study participants and/or their legal guardians.

### Nasal epithelial cell cultures, cell lines, and growth conditions

Primary human nasal epithelial cells (NECs) were obtained as previously described [6]. Briefly, NECs were obtained from healthy adult volunteers by gently stroking the inferior surface of the turbinate several times with a Rhino-Probe curette (Arlington Scientific, Arlington, TX), which was inserted through a nasoscope. This protocol was approved by the University of North Carolina School of Medicine Institutional Review Board for Biomedical Research. NEC were expanded to passage 2 in bronchial epithelial growth medium (BEGM, Cambrex Bioscience Walkersville, Inc., Walkersville, MD) and then plated on collagen-coated filter supports with a 0.4 µM pore size (Trans-CLR; Costar, Cambridge, MA) and cultured in a 1∶1 mixture of bronchial epithelial cell basic medium (BEBM) and DMEM-H with SingleQuot supplements (Cambrex), bovine pituitary extracts (13 mg/ml), bovine serum albumin (BSA, 1.5 µg/ml), and nystatin (20 units). Upon confluency, all-trans retinoic acid was added to the basolateral medium to establish air liquid interface (ALI) culture conditions (removal of the apical medium) to promote differentiation. Mucociliary differentiation was achieved after 18–21 days post-ALI. We obtained the Madin Darby canine kidney (MDCK) cell line from the American Type Culture Collection (ATCC, Manassas, VA). The BEAS-2B cell line was derived by transforming human bronchial cells with an adenovirus 12-simian virus 40 construct [47]. We obtained our BEAS-2B cells from the American Type Culture Collection (ATCC, Manassas, VA). BEAS-2B cells were grown in keratinocyte basal medium (KBM) supplemented with 30 µg/ml bovine pituitary extract, 5 ng/ml human epidermal growth factor, 500 ng/ml hydrocortisone, 0.1 mM ethanolamine, 0.1 mM phosphoethanolamine, and 5 ng/ml insulin.

The primary human NECs were exposed under ALI conditions to filtered air or 0.4 ppm ozone for 4 h in the exposure chambers operated by the U.S. Environmental Protection Agency, Environmental Public Health Division. In noted experiments, 10 mM of a cell-permeable form of reduced glutathione, glutathione-ethylester (GSH-ET) (Sigma-Aldrich St. Louis. Mo) or 1 µM of EGCG (Sigma-Aldrich St. Louis. Mo) was added to the basolateral medium 30 min prior to ozone exposure, similar to our previous studies [6], [46].

### Virus-like particle (VLP) entry assay

#### Construction of VLP expression plasmids

To generate the β-lactamase-M1 fusion expression plasmid (pCAGGS-β-lacM1 PR8) the influenza A/Puerto Rico/8/34/Mount Sinai (H1N1) M1 sequence was PCR amplified from the M1 expression vector pDZ-M (which was kindly provided by Dr. Adolfo Garcia-Sastre, Mount Sinai School of Medicine) and inserted into the pCAGGS vector [48], [49], [50]. The β-lactamase gene was PCR amplified from pcDNA3.1 and fused N-terminally to M1 within pCAGGS to create a modified β-lactamase-M1 fusion. In the modified β-lactamase, the first 24 amino acids were excluded to remove a secretion signal and His 24 was substituted with Asp to create an optimal Kozak consensus sequence. The pcDNA3.1-β-lactamase construct has been described previously [51]. The influenza A/Puerto Rico/8/34/Mount Sinai (H1N1) HA (pCAGGS-HA) and NA (pDZ-NA) over-expression vectors were generously provided by Dr. Aldolfo Garcia-Sastre and have been described previously [48], [49], [50], [52].

#### Production of VLPs

To generate influenza A/PR/8/H1N1 β-lactamaseM1VLPs (PR8 β-lacM1 VLPs), 293T cells were co-transfected with 3 µg each of pCAGGS-HA, pDZ-NA, and pCAGGS-β-lacM1 PR8 using FuGENE® HD (Roche Applied Science, Indianapolis, IN) according to manufacturer's instructions. The supernatant containing the VLPs were collect 48 h post-transfection and clarified of floating cell debris by centrifugation at 3,000 rpm for 10 min. The VLPs were concentrated once by low-speed centrifugation through an Amicon Ultra 100 kD centrifuge filter unit (Millipore; Billerica, MA), and the retentates were aliquoted and stored at −80°C.

#### VLP entry assay

The VLP entry assay was performed as previously described [6] with modifications. Briefly, target cells were exposed to filtered air or 0.4 ppm ozone for 4 h. 24 h post-exposure, VLPs were added to the apical surface in a total volume of 100 µl and incubated at 37°C for 3 h. The cells were washed twice with HBSS to remove unbound virus and infected cells were detected by using GeneBLAzer™ FRET *in vivo* cell-based assay system substrate CCF2-AM according to manufacturer's recommendations (Invitrogen). Samples were lysed in HBSS by freeze-thaw method repeatedly treated for 3 cycles (frozen in liquid nitrogen for 3 min and thawed in a 65°C water bath for 3 min). Viral entry was quantified by using the POLARstar OPTIMA plate reader (BMG LABTECH, Inc.). All experiments were performed in triplicate in three independent experiments.

### Infection with Influenza A

We used influenza A/Bangkok/1/79 H3N2 serotype (which was kindly provided by Dr. Melinda Beck, University of North Carolina). We also obtained influenza A/Malaya/302/1954 H1N1 serotype from the American Type Culture Collection (ATCC, Manassas, VA) for Western blot analysis of the cleavage products of the viral HA protein. Virus was propagated in 10-day-old embryonated hen's eggs. The virus was collected in the allantoic fluid and titered by 50% tissue culture infectious dose in Madin-Darby canine kidney cells and hemagglutination as described before [53]. Stock virus was aliquoted and stored at −80°C until use. Unless otherwise indicated, for infection about 5×10^5^ cells were infected with approximately 128 hemagglutination units (HAU) of influenza A, which resulted in approximately 10% of the cells being infected with influenza 24 h post-infection. Total RNA, total protein, basolateral supernatants, and apical washes were collected 24 h post-infection.

### Immunoblotting

Apical surface liquid was collected at 24 h post-exposure by washing the apical surface of the cells with HBSS and total protein concentrations were determined by Bradford protein assay (Bio-Rad). Cell lysates were prepared at 24 h post-infection in Passive Lysis Buffer (Promega, Madison, WI) with a protease inhibitor mixture (Cocktail Set III; Calbiochem, San Diego, CA) as well as phosphatase inhibitors (0.5 mM NaVO4, 1 mM β-glycerophophate) on ice for 30 min. After centrifugation, total protein concentrations were determined by Bradford protein assay (Bio-Rad). Both the apical supernatants and cellular lysates were subjected to 12% sodium dodecyl sulfate-polyacrylamide gel electrophoresis (SDS-PAGE) and transferred to nitrocellulose (Schleicher & Schuell Biosciences, Keene, NH). Proteins were detected using specific antibodies to HAT, TMPRSS2, and SPLI (1∶1,000; Santa Cruz Biotechnology, Santa Cruz, CA) or Influenza A virus hemagglutinin H1 antibody (1∶1,000; Abcam, Cambridge, MA). β-actin (1∶2,000; US Biological, Swampscott, MA), which was used as a loading control. Antigen-antibody complexes were stained with anti-rabbit or anti-mouse, horseradish peroxidase (HRP)-conjugated antibody (1∶2000, Santa Cruz Biotechnology) and detected with SuperSignal West Pico Chemiluminescent Substrate (Pierce, Rockford, IL). Densitometry was performed using a Fujifilm LAS-3000 imager (Fuji Film Global Tokyo, Japan).

### Influenza HA cleavage

Apical surface liquid was collected by washing the apical surface of the cells with HBSS and total protein concentrations were determined by Bradford protein assay (Bio-Rad). 50 µg of total protein were immunoprecipated overnight at 4°C with 200 ng of anti-TMPRSS2, anti-HAT, or IgG isotype as a control, followed by 1 h incubation with 50 µl of protein G-agrose beads (Pierce, Rockford, IL). Cleared supernatants were incubated with 50 ul of Influenza A/Malaya/302/1954 H1N1 for 30 min at 32°C. Virus was subjected to 12% sodium dodecyl sulfate-polyacrylamide gel electrophoresis (SDS-PAGE) and transferred to nitrocellulose (Schleicher & Schuell Biosciences, Keene, NH). Proteins were detected using specific antibodies to HAT, TMPRSS2, and SPLI (1∶1,000; Santa Cruz Biotechnology, Santa Cruz, CA) or Influenza A virus hemagglutinin H1 antibody (1∶1,000; Abcam, Cambridge, MA).

### Influenza virus titer

Influenza virus titers in apical washes were determined by 50% tissue-culture infectious dose (TCID50) in Madin Darby canine kidney (MDCK) cells and evaluated using agglutination of red blood cells as an indicator according to a modified protocol described before [54]. Briefly, MDCK cells grown in round-bottom 96-well plates were inoculated with virus-containing samples diluted in serum-free DMEM containing 20 µg/ml trypsin using log10 dilutions. After 3 days incubation, a suspension of human erythrocytes (0.5%) was added to each well. Wells exhibiting hemagglutination were considered positive and virus titers were expressed as log TCID_50_.

### Protease activity assay

A modification to the above protocol was performed to test secreted protease activity. In these experiments, NECs were exposed to either 0.4ppm of ozone or air for 4 h. 24 h post-exposure, apical supernatants were collected and protein concentrations were determined by Bradford protein assay. 100 µg of protein within the apical supernatant, which contained the endogenous secreted proteases, was incubated with 80 µl of virus for 30 min at room temperature. MDCK cells grown in round-bottom 96-well plates were inoculated with the above samples diluted in serum-free DMEM or serum-free DMEM containing 20 µg/ml trypsin, as a positive control, using 10-fold dilutions. After 3 days incubation, a suspension of human erythrocytes (0.5%) was added to each well. Wells exhibiting hemagglutination were considered positive and virus titers were expressed as log TCID_50_. 10 mM Phenylmethyl sulfonyl floride (PMSF) (Sigma-Aldrich St. Louis Mo) was used to inhibit trypsin protease activity in control samples.

### Protease inhibition assay

Recombinant human SLPI (rhSLPI) (R&D Systems Minneapolis MN) was added to serum-free DMEM containing 20 µg/ml trypsin and incubated for 60 min at room temperature prior to addition of 80 µl of virus. MDCK cells grown in round-bottom 96-well plates were inoculated with the above samples diluted in serum-free DMEM using 10-fold dilutions. After 3 days incubation, a suspension of human erythrocytes (0.5%) was added to each well. Wells exhibiting hemagglutination were considered positive and virus titers were expressed as log TCID_50_. All experiments were performed in triplicate in three independent experiments.

### RT-PCR

Total RNA was extracted using TRizol (Invitrogen) according to the supplier's instruction. First-strand cDNA synthesis and real-time RT-PCR was performed as described previously [46], [53] using commercially available primers and probes for HA (inventoried Taqman Gene Expression Assays) purchased from Applied Biosystems (Foster City, CA).

### Measurement of Lactate Dehydrogenase (LDH) and Interleukin-6 (IL-6) levels

NECs were exposed to filtered air or 0.4 ppm ozone for 4 h. 24 h post exposure, basolateral supernatants were collected for the measurement of LDH concentration according to the manufacturer's recommendations (Takara Bio Inc. Madison, WI). Released LDH concentration was expressed as optical density. Measurements of IL-6 concentration were determined according to the manufacturer's recommendations (BD Biosciences San Diego, CA). Released IL-6 concentration was expressed as pg/ml.

### SLPI trans-activation reporter assay

To measure trans-activation of the SLPI promoter, 1.2×10^5^ BEAS-2B were cotransfected with either 0.1 µg of SLPI-luciferase reporter plasmid (which was kindly provided by Dr. Rosalia Simmen, Arkansas Children's Hospital Research Institute) along with 0.02 µg of Thymidine kinase-*Renilla* luciferase by using Fugene 6 (Roche) according to the manufacturer's recommendations. After 24 h, cells were treated with 1 µM EGCG or DMSO. 8 h post-treatment cells were pelleted and lysed in passive lysis buffer (Promega, Madison, WI). The trans-activation activity was measured as luciferase light units as described previously [55]. All transfection experiments were performed in triplicate and normalized for transfection efficiency by using Renilla Luciferase.

### Statistical Analysis

Unpaired Student's t-test and One-way ANOVA were used for determination of statistically significant differences. The use of the term significant in text refers to a comparison of values for which p<0.05.

## Results

### Ozone exposure enhances viral replication

**Figure 1:**
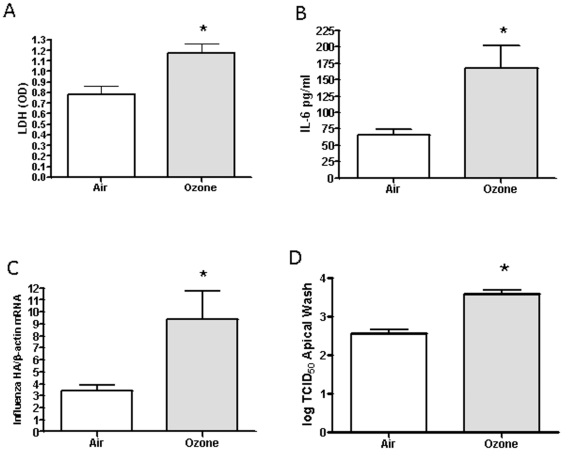
Exposure to ozone enhances influenza infection. Cultures of differentiated human epithelial cells were exposed to either 0.4ppm of ozone or air for 4 h. **A**) Basolateral supernatants collected 24 h post-exposure were analyzed for LDH levels. **B**) Basolateral supernatants collected 24 h post-exposure were analyzed for IL-6 levels. **C**) 24 h post-exposure, cells were infected with influenza A/Bangkok/1/79. Total RNA was isolated and subjected to RT-PCR to quantify Influenza *HA* transcripts and normalized to the expression of *β-actin*. **D**) 24 h post-exposure, cells were infected with influenza A/Bangkok/1/79. Apical supernatants collected 24 h post-infection were analyzed for determination of the viral titer indicated in log TCID_50_. NECs were obtained from six healthy volunteers (n = 6), and each experiment was performed in triplicate. Asterisk indicates statistical significance between test sample and control, * *p*<0.05.

Our group has recently demonstrated that oxidative stress increases susceptibility to influenza virus and that these responses may be mediated via increased viral entry [6], [46]. Therefore, we wanted to evaluate how exposure to ozone affects viral entry and replication. We chose to examine influenza infection in human NECs exposed to 0.4ppm ozone for 4 h, based on our group's recent *in vivo* [12], [56] and *in vitro* [9] studies. To assess the effects of ozone on NECs, we determined LDH and IL-6 levels in basolateral supernatants as markers of cytotoxicity and inflammation, respectively (Figure 1A and 1B). As expected, exposure to 0.4ppm of ozone for 4 h causes a significant increase in both inflammation and cytotoxicity [57]. Subsequently, 24 h post-exposure, cells were infected with influenza A/Bangkok/1/79 or a mock control. 24 h post-infection total RNA was subjected to real time RT-PCR to quantitate the influenza viral hemagglutinin transcript (HA) (Figure 1C). As depicted, we saw a significant increase in the amount of viral *HA* mRNA produced in the cells previously exposed to ozone as compared to the filtered air control. Similarly, by analyzing the apical washes for influenza viral titers 24 h post-infection, we saw a significant increase in viral titers in ozone exposed cells as compared to the control (Figure 1D). These results demonstrate that pre-exposure to ozone significantly increased viral replication in NECs.

### Ozone exposure does not affect cellular antiviral responses

**Figure 2:**
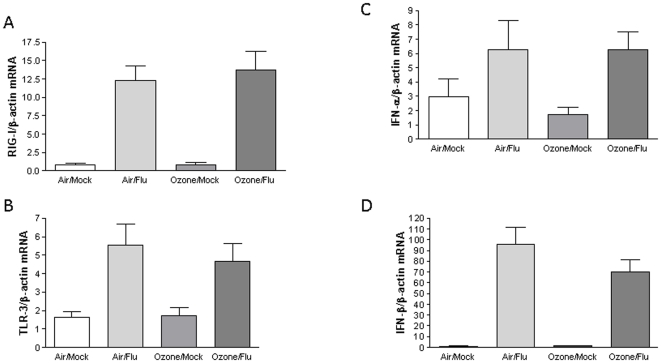
Exposure to ozone does not affect cellular antiviral responses. Cultures of differentiated human epithelial cells were exposed to either 0.4ppm of ozone or air for 4 h. 24 h post-exposure, cells were infected with influenza A/Bangkok/1/79. 24 h post-infection **A**) Total RNA was isolated and subjected to RT-PCR to quantify RIG-I transcripts and normalized to the expression of *β-actin*. **B**) TLR-3 transcripts and normalized to the expression of *β-actin*. **C**) IFN-α transcripts and normalized to the expression of *β-actin*. **D**) IFN-β transcripts and normalized to the expression of *β-actin*. NECs were obtained from six healthy volunteers (n = 6), and each experiment was performed in triplicate. Asterisk indicates statistical significance between test sample and control, * *p*<0.05.

To determine the potential mechanism(s) mediating the enhancement of viral replication following acute ozone exposure, we assessed whether innate antiviral immune response mediators were modulated. Specifically, we analyzed the expression of interferon-β (IFN-β), interferon-α (IFN-α), retinoic acid inducible gene I (RIG-I), and Toll-Like recptor-3 (TLR-3). Human NECs were exposed to ozone and 24 h post-exposure were infected with influenza A. 24 h post-infection total RNA was isolated and RT-PCR was performed to quantitate the amount of each cellular transcript. Ozone alone had no significant effect on mRNA expression in any of the four antiviral genes as compared to the air control (Figure 2A–D). As expected, we found substantial inductions of these four antiviral mediators 24 h post-infection with influenza A, but without significant differences in ozone exposed compared to control cells. These data demonstrate that exposure to ozone does not alter baseline expression of host antiviral immune response genes, and does not interfere with their induction in response to influenza infection.

### Antioxidant supplementation suppresses viral replication

**Figure 3:**
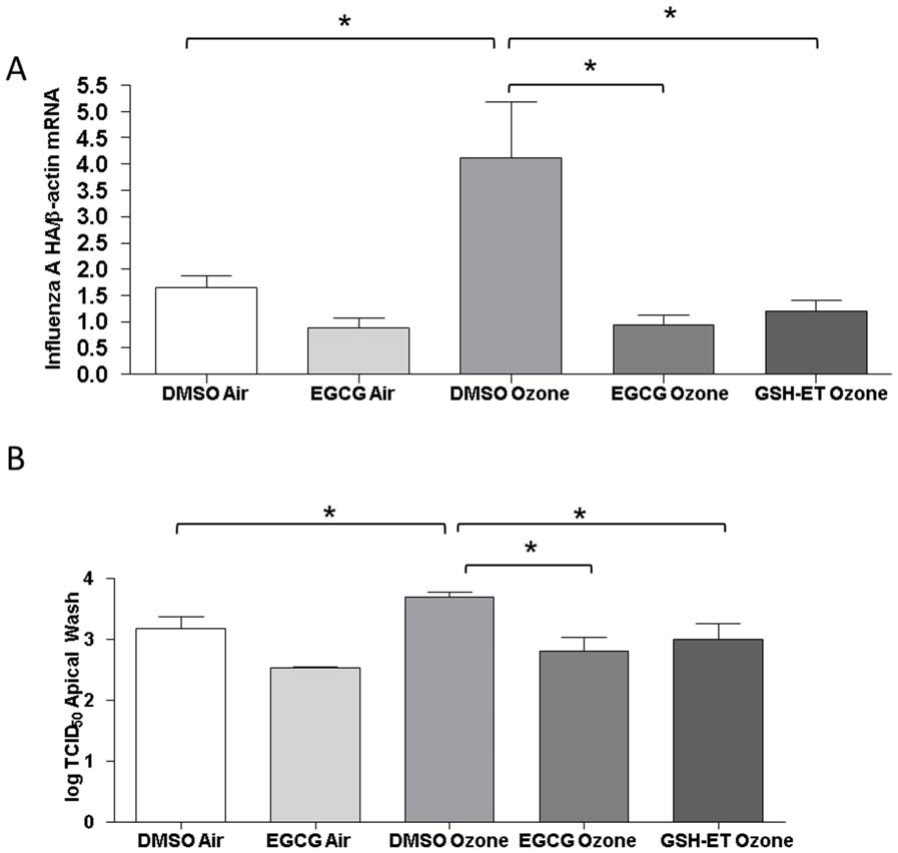
Antioxidant supplementation suppresses viral replication. Cultures of differentiated human epithelial cells were treated with EGCG, GSH-ET, or DMSO as a vehicle control for 30 min before exposure to either 0.4ppm of ozone or air for 4 h. **A**) 24 h post-exposure, cells were infected with influenza A/Bangkok/1/79. Total RNA was isolated and subjected to RT-PCR to quantify Influenza *HA* transcripts and normalized to the expression of *β-actin*. **B**) 24 h post-exposure, cells were infected with influenza A/Bangkok/1/79. Apical supernatants collected 24 h post-infection were analyzed for determination of the viral titer indicated in log TCID_50_. NECs were obtained from 6 healthy volunteers (n = 6), and each experiment was performed in triplicate. Asterisk indicates statistical significance between test sample and control, * *p*<0.05.

Since we have shown that ozone exposure did not abrogate antiviral responses, we wanted to determine if the increase in influenza virus infection is due to the oxidative stress pathway/mechanism induced by ozone exposure. To test this hypothesis, we treated our NECs with either a potent phase-II antioxidant inducer or a direct antioxidant. We have previously shown that oxidative stress increases susceptibility to influenza infection [6]. Previous reports have shown that *Nrf2* gene expression and protein expression can be induced via antioxidant supplementation [58], [59], [60], more specifically by the addition of the polyphenolic catechin, epigallocatechin-3-gallate (EGCG). This compound has the ability to induce *Nrf2* activation which in turn up-regulates the expression of multiple phase-II antioxidants [6], [61], [62]. Based on our previous published data [6], [46], we determined that 1 µM of EGCG or 10 mM of GSH-ET is sufficient to detect an increase in Nrf2 expression or counteract oxidative stress induced by diesel exhaust exposure in NECs. These concentrations also correlate with levels of EGCG that are achievable *in vivo* [63], [64], [65]. NECs were treated with either 1 µM EGCG, 10 mM GSH-ET, or DMSO as a vehicle control for 30 min prior to a 4 h 0.4ppm ozone exposure. 24 h post-ozone exposure cells were infected with influenza A virus and total RNA was isolated 24 h post-infection to determine the level of influenza HA transcripts by RT-PCR. As shown in Figure 3A, exposure to ozone significantly increases viral HA expression. Interestingly, the addition of either EGCG or GSH-ET significantly inhibited viral HA transcription as compared to the vehicle control. We again collected apical washes to perform viral titer assays. Our results, shown on a log scale, indicate that both EGCG and GSH-ET significantly reduced viral replication (Figure 3B). Taken together, these results demonstrate that antioxidants, either through Nrf2 activation (EGCG) or by direct addition of an antioxidant (GSH-ET), counteract the oxidative stress induced by ozone exposure and can suppress influenza virus replication in NECs.

### Ozone exposure increases viral entry

**Figure 4:**
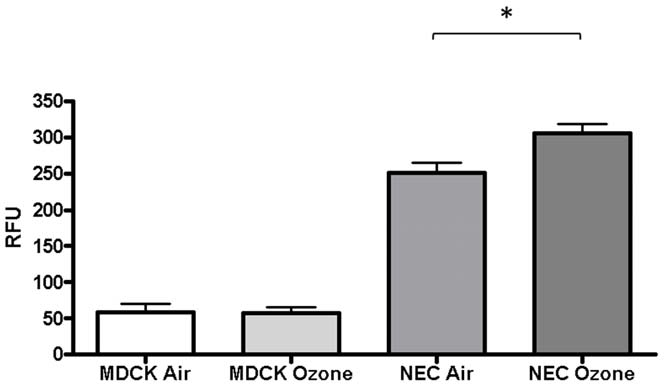
Ozone exposure increases viral entry. Cultures of differentiated human epithelial cells and MDCK cells used as controls, were exposed to either 0.4ppm of ozone or air for 4 h. 24 h post ozone exposure cells were infected with PR8 β-lacM1 VLP. At 4 h post-infection, cells were loaded with CCF2-AM substrate and assayed for viral entry. Data from a representative experiment performed in triplicate. Asterisk indicates statistical significance between test sample and control, * *p*<0.05.

Ozone not only triggers intracellular oxidative stress, but has also been shown to directly affect and modify the cellular lipid membrane [66], [67] and transmembrane molecules [68]. Since the influenza virion must first bind and enter the target cell prior to replication, we hypothesize that the increased susceptibility to influenza after ozone exposure most likely happens early in the virus lifecycle, upstream of viral replication. To determine which step(s) in the virus life cycle are affected by ozone exposure, we employed an enzymatic virus-like particle (VLP) assay. This assay quantitatively measures the amount of virus that enters the cells. Similar to our previous work [6], cells were infected with a VLP that only express the hemagglutinin (HA), neuraminidase (NA), and matrix (M) proteins along with a functional β-lactamase reporter fusion (PR8 β-lacM1 VLP) prior to loading with the fluorogenic substrate CCF2-AM . In cells in which the PR8 β-lacM1 VLP has entered, the CCF2-AM substrate will be cleaved, disrupting FRET of the substrate, and resulting in increased CCF2 emission at 447 nm. To determine the effects of ozone exposure on virus entry, NECs and the cell line MDCK as a control were exposed to air or ozone and 24 h post-exposure, cells were infected with PR8 β-lacM1 VLP. In this experiment, MDCK cells are used as a control cell line which does not produce proteases required to activate the viral HA protein. Figure 4 shows that exposure to ozone results in a significant increase in viral entry in the NECs alone. In summary, these data demonstrate that acute exposure to ozone enhances viral entry, consistent with the increased viral replication shown in Figure 1C.

### Ozone exposure disrupts host protease/antiproteases balance

**Figure 5:**
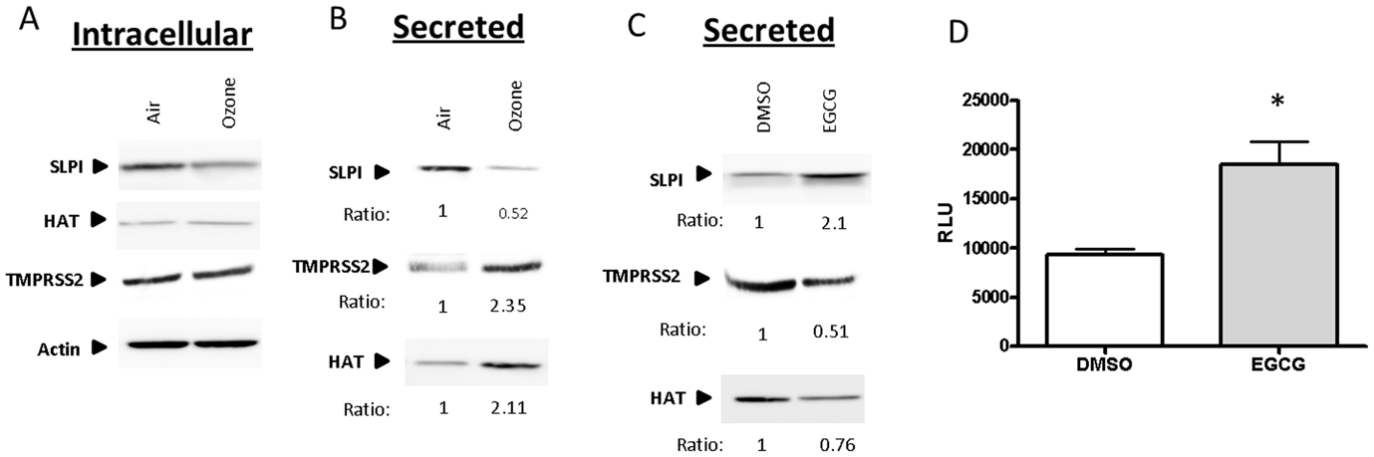
Ozone exposure modulates cellular protease and anti-protease levels. Cultures of differentiated human epithelial cells were exposed to either 0.4ppm of ozone or air for 4 h. 24 h post-exposure, samples were collected and subjected to Western blot analysis to detect SLPI, HAT, TMPRSS2, and β-actin. **A**) Total cellular lysates were analyzed for intracellular SLPI, TMPRSS2, and HAT protein expression by Western blot. Membrane was stripped and analyzed for β-actin as a loading control. **B**) 24 h post-exposure, apical supernatants were analyzed for secreted SLPI, TMPRSS2, and HAT protein levels. **C**) Cultures of differentiated human epithelial cells were treated with EGCG or DMSO as a vehicle control for 30 min before exposure to either 0.4ppm of ozone or air for 4 h. 24 h post-exposure, apical supernatants were analyzed for secreted SLPI, TMPRSS2, and HAT protein levels. **D**) Function activity of the SLPI promoter was determined using a dual-luciferase reporter assay. BEAS-2B cells were co-transfected with SLPI-Luc and TK-Renilla. Cells were treated with EGCG or DMSO as a vehicle control. Cells were harvested 8 h post-treatment and analyzed for luciferase expression. The values represent luciferase production from a representative experiment performed in triplicate. NECs were obtained from four healthy volunteers (n = 4). Densitometry was used to quantitate the amounts of protein, and the numbers below the gel indicate the ozone/air or EGCG/DMSO sample ratio.

As stated earlier, recent reports have identified two endogenous human trypsin-like serine proteases (TMPRSS2 and HAT) that possess the ability to cleave influenza virus *in vitro* [30], [32], [33], [69]. Viral HA proteolytic cleavage is required for viral fusion and entry into the host cell. Proteases are regulated by antiproteases, such as SLPI and to a lesser extent A1AT. Studies have shown that a disruption of the protease/antiprotease balance is a hallmark of numerous lung diseases and pathologies including COPD, emphysema, asthma, and cancer [40], [70], [71], [72]. Hennet et al, demonstrated a link between oxidative stress and protease expression which led to increased influenza infection in mice [39]. Linking this work with our current hypothesis, we investigated the protease/antiprotease balance on the epithelial surface in the context of an acute ozone exposure. For these experiments we exposed our NECs to either air or ozone for 4 h. 24 h post-exposure, both apical surface liquid and cell lysates were collected to characterize secreted/soluble and membrane-bound intracellular levels of HAT, TMPRSS2, and SLPI. Western blots revealed that intracellular levels of SLPI decreased slightly with ozone exposure, while HAT and TMPRSS2 expression remained relatively unchanged (Figure 5A). Although it is well known that SLPI is secreted and is present in normal human airway surface liquid, only recently was the transmembrane protease, HAT, found to be secreted in an *in vitro* overexpression system [33]. We found the levels of the secreted forms of HAT, TMPRSS2, and SLPI to be significantly changed by ozone exposure (Figure 5B). Under normal conditions, SLPI is in greater abundance than the proteases, yet 24 h post ozone exposure, we found a decrease in SLPI but a significant increase in both HAT and TMPRSS2. It is worth noting that the time at which these samples were taken corresponds to the exact time post ozone exposure when the cells were infected with influenza in the data shown in Figures 1, 2 and 3, thus representing the levels of protease and antiprotease present at the time of infection. To determine if disruption in the protease/antiprotease equilibrium is due to oxidative stress imposed by the ozone exposure, we treated our NECs with the potent Nrf2 inducer EGCG prior to ozone challenge. Figure 5C shows that EGCG did increase levels of SLPI which corresponds with a decrease in protease expression. Finally, Figure 5D demonstrate that the addition of 1 µM EGCG significantly increases the trans-activation of the SLPI promoter which correlates with the increase in SLPI protein expression displayed in Figure 5C. Taken together, these results indicate that ozone-induced oxidative stress disrupts the protease/antiprotease balance, in favor of protease activity. Furthermore, we demonstrate that the equilibrium can be restored with the induction of Nrf2 via antioxidant supplementation resulting in an increase in the antiprotease SLPI.

### Secreted proteases are functional for hemagglutinin cleavage

**Figure 6:**
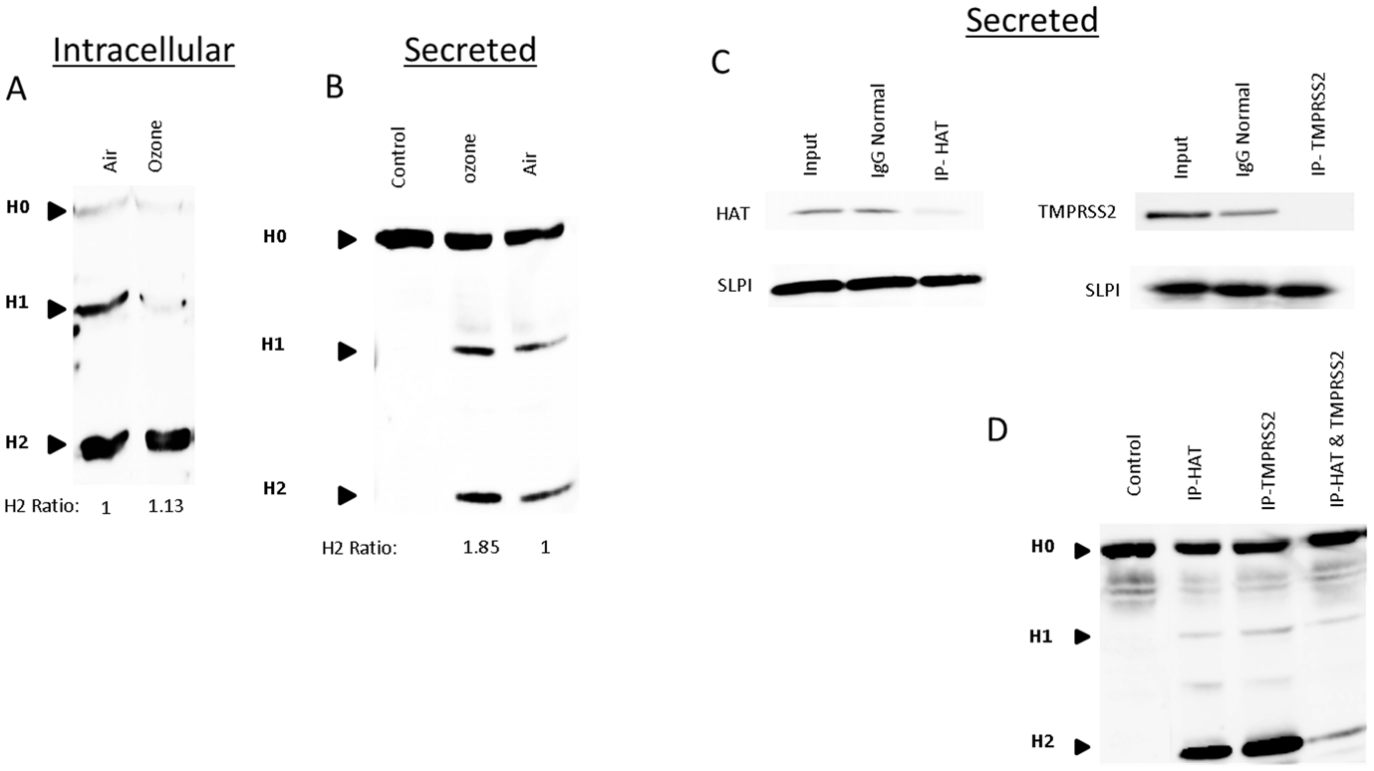
Secreted proteases are functional for Hemagglutinin cleavage. Cultures of differentiated human epithelial cells were exposed to either 0.4ppm of ozone or air for 4 h. A) 24 h post-exposure, cells were infected with influenza A/Malaya/302/1954. 24 h post-infection, total cellular lysates were analyzed for intracellular cleavage of the viral HA protein by Western blot. B) 24 h post-exposure, apical supernatants were collected and incubated with influenza A/Malaya/302/1954. Cleavage of the viral HA protein was analyzed by Western blot. C) Cultures of differentiated human epithelial cells were exposed to either 0.4ppm of ozone or air for 4 h. 24 h post-exposure, apical supernatants were immunoprecipitated (IP) with anti- HAT, anti- TMPRSS2, or an isotype control (IgG). Protein levels were analyzed by Western blot. D) Apical supernatants of differentiated human epithelial cells, were immunoprecipitated (IP) with anti- HAT, anti- TMPRSS2, or an isotype control (IgG) followed by incubation with influenza A/Malaya/302/1954. Cleavage of the viral HA protein was analyzed by Western blot. NECs were obtained from four healthy volunteers (n = 4). Densitometry was used to quantitate the amounts of cleaved H2 protein, and the numbers below the gel indicate the ozone/air ratio.

We characterized the cleavage products of the viral HA protein to determine if the proteases produced by acute ozone exposure can cleave an intact influenza virion. We first investigated where HA cleavage takes place. NECs were exposed to air or ozone and 24 h post-exposure, cells were infected with Influenza A/Malaya/302/1954 H1N1. The cell lysates were analyzed by Western blotting for detection of the HA cleavage products. Figure 6A shows that the majority of the viral HA protein within the cell is cleaved and there is not a significant change with ozone exposure. In addition, Western blot analysis of apical washings incubated with virus showed that HA0 was cleaved and that there was an increase in the cleavage of HA from the ozone exposed cells as measured by densitometry. Quantification of the H2 cleavage products demonstrated that ozone exposure leads to enhanced proteolytic activation of the virion. This data demonstrate that HA cleavage can take place in both the intra- and extracellular environment and that ozone exposure increases the proteolytic activation of the virus in the airway (Figure 6B). To characterize the function of TMPRSS2 and HAT in HA cleavage, we depleted the proteases via immunoprecipitation prior to addition of the virus. Figure 6C shows the apical washes from NECs that were immunoprecitipated with anti-HAT, anti-TMPRSS2, and IgG as a control. The cleared supernatants were incubated with Influenza A/Malaya/302/1954 H1N1. As shown in Figure 6D, there is little difference in the cleavage of the HA protein when the protease are depleted separately, but we demonstrate a significant decrease in the cleavage of HA when both proteases were removed. This data show that the protease(s) found in the apical surface liquid are able to cleave an intact influenza A virion. In addition we show that there are additional proteases secreted into the airway liquid space that are able to activate the virus.

### Secreted proteases are functional and can activate Influenza A virions to propagate a viral infection

**Figure 7:**
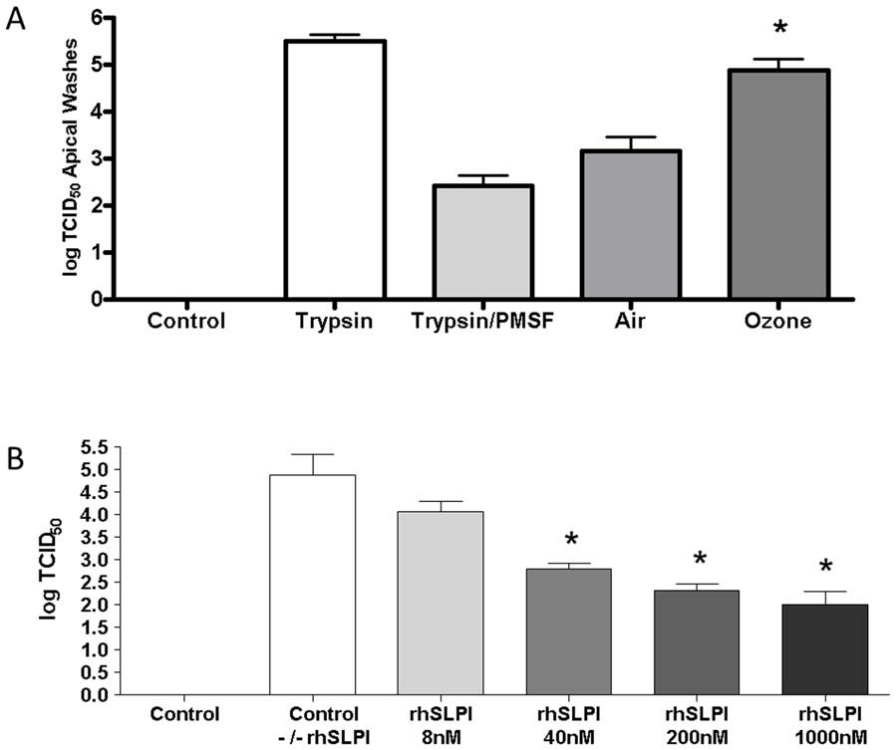
Secreted proteases are functional and can activate virions to propagate a viral infection. **A**) Cultures of differentiated human epithelial cells were exposed to either 0.4ppm of ozone or air for 4 h. 24 h post-exposure apical supernatants were incubated with influenza A/Bangkok/1/79 for 30 min, and then analyzed for determination of the viral titer indicated in log TCID_50_. **B**) Increasing amounts of rhSLPI (8 nM, 40 nM, 200 nM, 1000 nM) were incubated with media containing 20 µg/ml trypsin for 60 min, then analyzed for determination of the viral titer indicated in log TCID_50_. NECs were obtained from four healthy volunteers (n = 4), and each experiment was performed in triplicate. Data from a representative experiment performed in triplicate. Asterisk indicates statistical significance between test sample and control, * *p*<0.05.

Although we show that SLPI, HAT, and TMPRSS2 are secreted by NECs, the question remains as to whether they are functional. To test functionality and the role they play in a viral infection, we employed a modified infectivity viral titer assay. These experiments are very similar to the viral titer assays mentioned earlier, but with a few modifications. Similar to the assays before, we utilized the well-characterized MDCK cell line which does not produce the proper proteases to activate the influenza virion, which can be overcome by the addition of exogenous trypsin. In contrast to the previous titer experiments above, we did not add exogenous trypsin to enable multicycle replication of influenza virus in these cells. These experiments allowed us to test if secreted proteases present in the apical surface liquid from NECs exposed to air or ozone were able to facilitate multiple rounds of viral replication in MDCKs. We show that the secreted proteases present in the apical supernatants from NECs are functional and can propagate viral entry and replication. We also demonstrate that the ozone-exposed NEC supernatants displayed significantly greater infection kinetics than the air control (Figure 7A). This correlates with the increase in protease expression post ozone exposure (Figure 5B), viral entry (Figure 4), and viral replication (Figure 1C and 1D). In addition we show that this is a protease-mediated event, since the addition of a known serine protease inhibitor, PMSF, significantly inhibits viral replication.

To demonstrate that SLPI has the ability to protect airway epithelium from influenza infection by inhibiting proteolytic cleavage, we incubated various amounts of recombinant human SLPI (rhSLPI) to our traditional viral titer assay. Briefly, we incubated our viral titer media, serum-free DMEM containing 20 µg/ml of exogenous trypsin, with varying amounts of rhSLPI prior to addition of influenza. As shown in Figure 7B, addition of rhSLPI significantly inhibits multicycle viral replication. We conclude that the addition of rhSLPI directly inhibits trypsin-mediated cleavage of viral HA leading to decreased infection and replication. Taken together, these data demonstrate that exposure to ozone disrupts the protease/antiprotease balance resulting in greater secretion of endogenous proteases leading to increased viral cleavage/activation culminating in enhanced viral entry and replication.

## Discussion

Numerous epidemiological studies link increased ambient ozone levels with respiratory viral infections [1], [73], [74]. However, the role of ozone exposure in viral replication and pathogenesis remains to be fully defined. Because ambient ozone is a major source of oxidative stress in the airway epithelium and this is the primary site for influenza infections, it is important to determine the role ozone exposure plays in viral infections. Although it has been shown that ozone exposure can modulate many aspects of the immune response [8], how these alterations influence a viral infection have yet to be elucidated. Recent studies have demonstrated the importance of the protease/antiprotease balance in the context of normal lung homeostasis, and have shown that oxidative stress can disrupt this delicate equilibrium [40], [75]. It has been well documented that many lung pathologies are dependent on the regulatory interplay between oxidative stress and protease expression [40], [70], [71]. Although there is speculation that Mexico City's increased influenza-related morbidity and mortality rates were due to exposures to higher levels of ozone, little research has been done looking at the effects of pre-exposure to ozone in the context of an influenza infection in humans. Herein, we demonstrate that acute ozone exposure disrupts the protease/antiprotease balance resulting in increased secreted protease expression in primary human epithelial cells, resulting in increased HA cleavage and activation of the influenza virion, which correlated with enhanced viral entry and replication.

Numerous host cell-dependent factors can affect and control influenza virus attachment and uptake by: (i) proteolytic cleavage of viral HA by host cell-derived serine proteases [30], [33], (ii) host cell derived innate immune defense molecules aimed at inhibiting the infectious virions [25], [76], and (iii) antiviral mediators limiting viral replication and shedding of virus particles [6], [52]. We first focused on potential effects of ozone on host antiviral defense responses. Our data showed that exposure to ozone elicited the typical pro-inflammatory and cellular damage responses as seen with increased release of IL-6 and LDH. We found that the same exposure regimen also resulted in significant increase in viral replication. We initially hypothesized that this could be due to a disruption in one of the cellular antiviral response mechanism/pathways, and assessed the transcription levels of 4 classical antiviral mediators; RIG-I, IFN-α, IFN-β, and TLR-3. Similar to our previous studies using exposure to diesel exhaust [46], we found that ozone did not decrease expression of these mediators or their response when challenged with influenza. In our previous study [46], we found that exposure to an oxidant pollutant increased influenza virus attachment and/or entry, but did not distinguish between these two steps in the viral infection cycle. Therefore, we focused our attention on steps upstream of viral replication to identify mechanisms by which oxidants, such as ozone, affect influenza infectivity.

To dissect specific points in the virus life cycle upstream of viral replication that could determine the role ozone exposure plays in viral susceptibility, which ultimately dictates viral pathogenesis and outcome, we utilized our previously described enzymatic virus-like particle (VLP) assay [6]. Our data demonstrated that ozone exposure significantly increased influenza virus entry. This is consistent with our previous work showing that exposure to other oxidant pollutants and suppression of Nrf2 increases influenza virus entry [6], [46]. Similar to these previous studies, data shown here demonstrated that the mechanism(s) through which ozone exposure alters influenza virus entry is mediated by oxidative stress. Since ozone directly interacts with the apical surface of the respiratory epithelium we hypothesize that ozone either directly, or via the induction of biologically active ozone reaction products, modifies either the expression or function of surface proteins, as previously been demonstrated for human surfactant protein A (SP-A) [77]. Among the most prominent proteins on the apical surface potentially regulating infection are the proteases. To date, at least five different proteases have been identified in the airways of animals and humans [78]. Despite many years of effort, the exact protease(s) that activates influenza has yet to be identified. Numerous groups have reported that the family of type II transmembrane serine proteases and trypsin-like proteases are responsible for viral cleavage [26], [30], [33], [79]. Previous studies have examined these proteases in the context of influenza infections, specifically TMPRSS2 and HAT. Although these studies demonstrated that these proteases are capable of cleavage, the experiments were performed in cell lines such as MDCKs [33] or the colorectal adenocarcinoma cell line Caco-2 [32], neither of which are natural target cells for influenza. Since the cellular protease determines the tropism as well as efficiency of viral replication, we utilized fully differentiated human primary nasal epithelial cells (NECs) in this study, a natural host cell for influenza virus. We show that HAT and TMPRSS2 are not only present, but secreted from NECs (Figure 5B), and that ozone exposure enhances the release of the proteases into the apical compartment. This is consistent with reports showing that oxidative stress increases cellular protease activity [38]. Since proteases in the upper airway are inhibited by SLPI [78], we focused on this antiprotease and its expression in relation to HAT and TMPRSS2. Similar to previous reports, we report that SLPI is detected in the secreted airway and oxidative stress reduces the expression and function of the protein (Figure 5B) [35], [80]. These data are consistent with our hypothesis that influenza virions can become activated in the airway surface liquid prior to binding to the epithelial surface. Virion activation in the airway prior to binding would eliminate the need for a membrane-bound protease thus broadening viral tropism from airway epithelium, that contain the necessary proteases, to any mammalian cell, since *2,6*-linked sialic acid is expressed by most mammalian cells ranging from lymphoid to glial cells [81], [82].This could explain the increased morbidity and mortality associated with the Mexico City 2009 H1N1 pandemic which resulted in increased infection of multiple cell types including distal airway epithelium and immune cells including alveolar macrophages [83], [84] as compared to previous seasonal outbreaks.

EGCG has been shown to have potent antioxidant capabilities; in part by inducing the expression of a number of antioxidant enzymes [85]. *In vitro* and *in vivo* studies have shown that this supplement induces the expression of phase II antioxidant genes such as GSTM1, which was associated with Nrf2-ARE signaling [58], [86]. Whether and how activation of antioxidants expression is involved in the potential antiviral effects of EGCG is not known. Previous studies have indicated that it directly binds influenza virus and therefore prevents attachment and entry into host cells [63], [87]. However, these studies were conducted in MDCK cells, which are not a natural host cell for influenza and require addition of exogenous proteases to achieve viral entry [63]. Iizuka et al, demonstrated that Nrf2-knockout mice display protease/antiprotease imbalance leading to increased susceptibility to cigarette smoke-induced emphysema [37]. In addition, the same group showed that Nrf2 expression exerts a protective effect through the transcriptional activation of antiproteases [88]. Our data demonstrate that supplementation of differentiated nasal epithelial cells with EGCG from the basolateral side (to eliminate direct interaction with the virus during infection) significantly increases SLPI production (Figure 5C). It is worthy to note that we show a direct inverse relationship between the levels of secreted SLPI and secreted protease expression, suggesting a potential novel antiviral mechanism by which EGCG could be protective against influenza infections.

We next determined that the secreted proteases are functional. We demonstrate that proteases present in the apical surface liquid were able to activate the virus (Figure 6B) and produce multiple rounds of viral replication (Figure 7A). In addition, apical supernatants from ozone-exposed epithelial cells had significantly higher viral titers, which correlate with the increased protease expression displayed in Figure 5B. In the final experiment we utilized rhSLPI and show a dose dependent protective response (Figure 7B). This demonstrates that that the antiprotease SLPI has a protective affect against viral activation and replication.

In conclusion, this is the first study to demonstrate that secreted proteases from primary human respiratory nasal epithelium proteolytically activate influenza virions and that exposure to a common ambient air oxidant pollutant increases these effects. We speculate that individuals with increased airway oxidative stress, whether due to an underlying medical condition or exposure to high levels of air pollutants, would develop disruption of the epithelial protease/antiprotease balance and become more susceptible to infections with viruses like influenza, SARS-CoV, and metapneumovirus. Disruption in the protease/antiprotease balance has been described in several respiratory diseases including COPD, emphysema, and asthma [70], [71], [89]. This mechanism could be exploited as a novel anti-viral therapy in combination with conventional antiviral treatments. Antiproteases, such as SLPI appear to protect the respiratory epithelium from influenza virus infection and nutritional supplements may increase the antiprotease:protease ratio. Taken together, the data shown here provide further support to the concept that targeting HA-activating proteases and decreasing oxidative stress to arrest viral infection and spread in conjunction with antiviral agents is a promising potential therapeutic intervention approach against influenza.
